# Persistent *Campylobacter jejuni* enterocolitis after CAR T-cell therapy in a patient with hypogammaglobulinemic follicular lymphoma

**DOI:** 10.1128/asmcr.00047-26

**Published:** 2026-08-20

**Authors:** Dina Oulkadi, Florian Van Oers, Leen Vanden Driessche, Sarah Vandamme, Veerle Matheeussen, Célestin Mairesse, Delphine Martiny, Sébastien Anguille, Thomas Demuyser

**Affiliations:** 1 Department of Clinical Microbiology, Antwerp University Hospital, Edegem, Belgium; 2 Division of Hematology and Center for Cell Therapy & Regenerative Medicine, Antwerp University Hospital, Edegem, Belgium; 3 Faculty of Medicine and Health Sciences, Laboratory of Experimental Hematology, Vaccine & Infectious Disease Institute (VAXINFECTIO), University of Antwerp, Antwerp, Belgium; 4 Department of Microbiology, Laboratoire Hospitalier Universitaire de Bruxelles-Universitair Laboratorium Brussel (LHUB-ULB), Brussels, Belgium; 5 Belgian National Reference Centre for Campylobacter (LHUB-ULB) 832616 , Brussels, Belgium; 6 Department of Bioscience Engineering, Laboratory of Applied Microbiology and Biotechnology, University of Antwerp, Antwerp, Belgium; Pattern Bioscience, Austin, Texas, USA

**Keywords:** *Campylobacter jejuni*, persistent infection, hypogammaglobulinemia, CAR T-cell therapy

## Abstract

**Background:**

*Campylobacter jejuni* is a leading cause of bacterial gastroenteritis worldwide and typically results in self-limiting enterocolitis in immunocompetent individuals. In contrast, immunocompromised patients are at risk for severe, recurrent, or persistent infection. Chimeric antigen receptor (CAR) T-cell therapy targeting CD19 induces prolonged B-cell aplasia and hypogammaglobulinemia, predisposing patients to atypical infections. We report the first case of persistent and disseminated *C. jejuni* infection following CD19-directed CAR T-cell therapy, confirmed by whole-genome sequencing.

**Case Summary:**

A 68-year-old man with relapsed follicular lymphoma underwent autologous CD19-directed CAR T-cell therapy and achieved complete remission. Pre-existing hypogammaglobulinemia worsened after treatment. Eighteen months later, he developed recurrent bacteremia and chronic enterocolitis caused by *C. jejuni*. Over the following year, he experienced severe watery diarrhea, 20 kg weight loss, and disseminated infection with a prosthetic hip abscess. More than 10 stool cultures remained positive despite multiple antibiotic regimens and two fecal microbiota transplantations. Whole-genome sequencing demonstrated a single clonal strain with minimal variation, confirming persistent infection rather than reinfection. Microbiological clearance was achieved after 8 weeks of oral gentamicin, followed by 2 weeks of intravenous meropenem and azithromycin, with sustained negative cultures and PCR.

**Conclusion:**

Persistent and disseminated *C. jejuni* infection can occur in patients with hypogammaglobulinemia after CD19-directed CAR T-cell therapy. Whole-genome sequencing may help distinguish persistence from reinfection. Standard immunoglobulin replacement may be insufficient due to limited IgA and IgM, and prolonged combination antimicrobial therapy may be required for durable remission.

## INTRODUCTION

*Campylobacter jejuni* is a microaerophilic gram-negative bacillus and a leading cause of bacterial gastroenteritis worldwide (1, 2). It is frequently isolated from human fecal cultures, with transmission occurring mainly through contaminated poultry or dairy products (1, 2). In healthy individuals, infection typically manifests as a self-limiting enterocolitis characterized by diarrhea, abdominal pain, and fever that resolves within a week (3).

In contrast, immunocompromised patients face a higher risk of severe, recurrent, or persistent *Campylobacter* infections (1, 3). Hypogammaglobulinemia, in particular, has been associated with recurrent enteritis due to impaired mucosal antibody-mediated immunity (3, 4). Chimeric antigen receptor (CAR) T-cell therapy has transformed the treatment of B-cell malignancies and multiple myeloma but frequently leads to hypogammaglobulinemia through B-cell depletion (5).

Here, we describe the first reported case of persistent *C. jejuni* enterocolitis and disseminated infection in a patient with hypogammaglobulinemia following CD19-targeted CAR T-cell therapy.

## CASE PRESENTATION

The case concerns a patient with a history of relapsed/refractory B-cell non-Hodgkin lymphoma (follicular lymphoma subtype). In October 2022, at 68 years of age, the patient underwent autologous CD19 CAR T-cell therapy as third-line treatment within a clinical trial. The infusion was uneventful, and a complete response was achieved 3 months later. Prior to CAR T-cell infusion, the patient already exhibited hypogammaglobulinemia (IgG ~4 g/L, reference level [RL]: 6.5–16 g/L). This further declined to ~2 g/L by January 2023, prompting initiation of monthly intravenous immunoglobulin (IVIG) replacement therapy. Serum IgG levels fluctuated in accordance with IVIG administration, whereas IgA (RL: 0.4–3.5 g/L) and IgM levels (RL: 0.5–3 g/L) remained persistently below the limit of detection by both turbidimetric and nephelometric assays.

**Fig 1:**
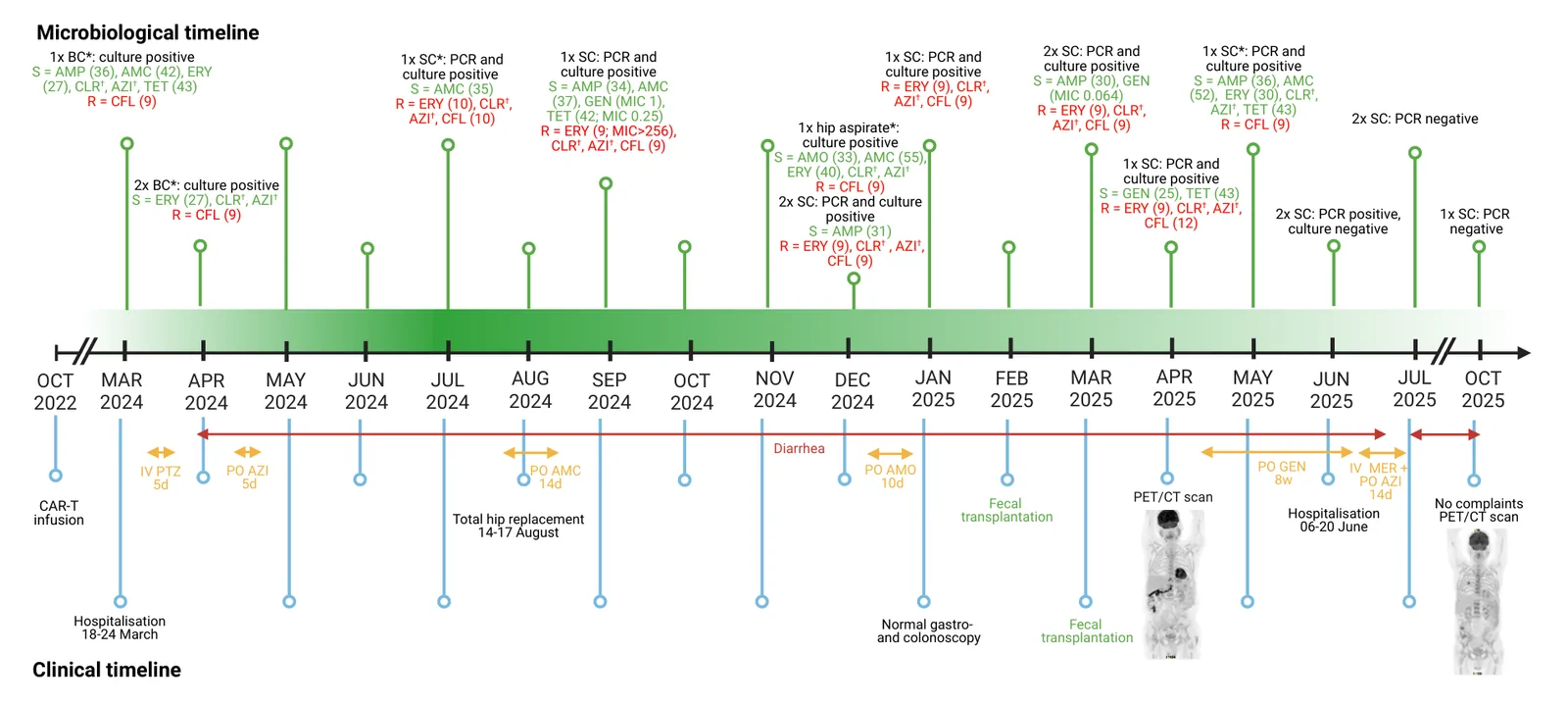
Microbiological and clinical timeline. Microbiological timeline showing sequential *C. jejuni* cultures and associated phenotypic resistance profiles. The clinical timeline below outlines diarrhea duration, antibiotic therapy, fecal transplantation, and PET/CT imaging. Zone diameters (mm) and/or MIC values (µg/mL) are reported in parentheses for each antibiotic; values indicate zone diameters unless stated as MIC. EUCAST breakpoints were applied for ciprofloxacin (zone diameter: S ≥ 50 mm, R < 26 mm), erythromycin (zone diameter: S ≥ 20 mm, R < 20 mm; MIC: S ≤ 4 µg/mL, R > 4 µg/mL), and tetracycline (zone diameter: S ≥ 30 mm, R < 30 mm; MIC: S ≤ 2 µg/mL, R > 2 µg/mL). Susceptibility to clarithromycin and azithromycin was inferred from erythromycin susceptibility (indicated by “†”). For ampicillin/amoxicillin (zone diameter: S ≥ 14 mm, R < 14 mm), amoxicillin–clavulanic acid (zone diameter: S ≥ 14 mm, R < 14 mm), and gentamicin (zone diameter: S ≥ 17 mm, R < 17 mm; MIC: S ≤ 2 µg/mL, R > 2 µg/mL), CA-SFM breakpoints were used. An asterisk (*) indicates that the *C. jejuni* strain was subjected to whole-genome sequencing (WGS) at the Belgian National Reference Center (NRC) for Campylobacter. AMP/AMO, ampicillin/amoxicillin; AMC, amoxicillin-clavulanate; AZI, azithromycin; BC, blood culture; CFL, ciprofloxacin; CLR, clarithromycin; ERY, erythromycin; GEN, gentamicin; IV, intravenous; PO, per os; SC, stool culture; S, sensitive; R, resistant; TET, tetracycline.

In March 2024, 1.5 years after CAR-T treatment, he was admitted to the hospital with fever and abdominal pain (Fig. 1). Symptoms resolved after 5 days of empiric intravenous piperacillin-tazobactam (4 g/500 mg four times daily). After 5 days of incubation, one of four blood culture sets yielded *C. jejuni*, showing resistance only to ciprofloxacin. One month later, blood cultures again revealed *C. jejuni* with the same resistance profile. Contrary to the first episode, he developed diarrhea and abdominal cramps. These symptoms improved after oral azithromycin treatment (500 mg once daily for 5 days). From July 2024 onward, he experienced recurrent diarrhea, with more than 10 stool cultures positive for *C. jejuni* over the course of a year. Multiple antibiotics were administered according to differing resistance profiles, but durable eradication was not achieved.

Clinically, he suffered from chronic watery diarrhea up to 15 times daily, accompanied by cramps, bloating, loss of appetite, and almost 20 kg weight loss, significantly impacting his quality of life. Additionally, in December 2024, he presented to the emergency department with fever and right hip pain. Evaluation revealed a hip abscess at the site of a total hip replacement performed 4 months earlier. Culture of the abscess aspiration again grew *C. jejuni*, while blood cultures were negative. The abscess was drained and successfully treated with oral amoxicillin (500 mg three times daily for 10 days).

Endoscopic evaluation of the colon and stomach/duodenum was unremarkable, and biopsy of the colon excluded cytomegalovirus reactivation. Since antibiotics showed limited effect, fecal microbiota transplantation was performed twice (February and March 2025). Improvement of the diarrhea was only short, and persistent *C. jejuni* colonization could still be found on stool culture and PCR. Remarkably, PET/CT scan performed in April 2025 confirmed sustained lymphoma remission but showed hypermetabolic uptake in the small intestine at the hepatic flexure.

Literature review identified a case of chronic *C. jejuni* in an immunodeficient patient (6) successfully treated with oral gentamicin (80 mg four times daily for 8 weeks). Based on this, oral gentamicin therapy was initiated in April 2025. After 4 weeks, diarrhea frequency improved modestly, but stool cultures remained positive. At week 8, stool culture became negative for the first time, but PCR was still positive. However, at the same time, he deteriorated clinically with malaise, poor oral intake, and worsening diarrhea, necessitating hospitalization. Gentamicin was stopped and replaced with intravenous meropenem (1 g three times daily) plus azithromycin (1 g daily) for 14 days.

Following this intervention, diarrhea resolved for the first time in nearly a year. Stool culture and PCR became negative for *C. jejuni*, with confirmation in two additional samples 1 month later. Subsequent intermittent diarrhea episodes remained *C. jejuni*-negative and were managed symptomatically with loperamide. By October 2025, the patient reported normalized stool and improved daily functioning. PET/CT imaging showed normalization of the intestines with a persistent complete metabolic response.

Phenotypic susceptibility testing was performed on each isolated strain. In this case, ciprofloxacin resistance was constant, while macrolide resistance fluctuated. Amoxicillin, amoxicillin–clavulanic acid, tetracycline, and gentamicin remained susceptible throughout.

To further characterize these phenotypic findings at the genomic level, five *C. jejuni* isolates underwent WGS at the Belgian National Reference Center (NRC) for Campylobacter, with virulence and resistance profiling using VFDB (v2025-10-10) and AMRFinderPlus (v2024-12-18.1) databases (7, 8), respectively, and sequence typing via PubMLST (9). WGS demonstrated that all five isolates belonged to clonal complex CC-443 (sequence type, ST-6522), with SNP and cgMLST analyses showing only two allelic differences between the earliest and latest strains. The genomes contained more than 90 virulence-associated genes and multiple resistance determinants (Table 1).

**TABLE 1:** Antimicrobial susceptibility and resistance determinants in sequential *C. jejuni* isolates^*a*^

Date of isolation	Site of isolation	Species	Phenotypic susceptibility	Resistance determinants for β-lactams (% identity)	Resistance determinants for macrolides (% identity)	Resistance determinants for quinolones (% identity)	Resistance determinants for tetracyclines (% identity)
19/03/2024	Blood	*C. jejuni*	S = AMP (36), AMC (42), ERY (27), CLR^*b*^, AZI^*b*^, TET (43) R = CFL (9)	*blaOXA-576* (100)	*rplV_A103V* (97, 87)	*gyrA_T86I* (99, 42)	*tet(O)* (99, 68)
16/04/2024	Blood	*C. jejuni*	S = ERY (27), CLR^*b*^, AZI^*b*^ R = CFL (9)	*blaOXA-576* (100)	*rplV_A103V* (97, 87)	*gyrA_T86I* (99, 42)	*tet(O)* (99, 68)
23/07/2024	Stool	*C. jejuni*	S = AMC (35) R = ERY (10), CLR^*b*^, AZI^*b*^, CFL (10)	*blaOXA-576* (100)	*rplV_A103V* (97, 87)	*gyrA_T86I* (99, 42)	*tet(O)* (99, 68)
11/12/2024	Hip aspirate	*C. jejuni*	S = AMO (33), AMC (55), ERY (40), CLR^*b*^, AZI^*b*^ R = CFL (9)	*blaOXA-576* (100)	*rplV_A103V* (97, 87)	*gyrA_T86I* (99, 42)	*tet(O)* (99, 68)
16/05/2025	Stool	*C. jejuni*	S = AMP (36), AMC (52), ERY (30), CLR^*b*^, AZI^*b*^, TET (43) R = CFL (9)	*blaOXA-576* (100)	*rplV_A103V* (97, 87)	*gyrA_T86I* (99, 42)	*tet(O)* (99, 68)

^*a*^ Zone diameters (mm) are reported in parentheses after each antibiotic. EUCAST breakpoints were applied for ciprofloxacin (zone diameter: S ≥ 50 mm, R < 26 mm), erythromycin (zone diameter: S ≥ 20 mm, R < 20 mm), and tetracycline (zone diameter: S ≥ 30 mm, R < 30 mm). For ampicillin/amoxicillin (zone diameter: S ≥ 14 mm, R < 14 mm) and amoxicillin–clavulanic acid (zone diameter: S ≥ 14 mm, R < 14 mm), CA-SFM breakpoints were used. AMP/AMO, ampicillin/amoxicillin; AMC, amoxicillin-clavulanate; AZI, azithromycin; CFL, ciprofloxacin; CLR, clarithromycin; ERY, erythromycin; GEN, gentamicin; S, sensitive; R, resistant; TET, tetracycline.

^*b*^ Susceptibility to clarithromycin and azithromycin was inferred from erythromycin susceptibility.

## DISCUSSION

To reduce infection risk, patients typically receive monthly IVIG substitution. Standard preparations contain mainly IgG and are low in IgA and IgM. However, similar cases described in primary hypogammaglobulinemia highlight the role of humoral immunity in controlling *Campylobacter* spp. Secretory IgA provides mucosal defense, though selective IgA deficiency is not clearly linked to increased *Campylobacter* spp. infection (3). Serum IgM appears more important, as it enhances complement-mediated phagocytosis about 200–1,000-fold more effectively than IgG (10, 11). Absence of both IgA and IgM thus predisposes to persistent colonization of *Campylobacter* spp. (3, 10).

Borleffs et al. (10) described two primary hypogammaglobulinemic patients with chronic *C. jejuni* infection unresponsive to antibiotics and IgG-based IVIG who improved with Pentaglobulin, an IgA/IgM-enriched IVIG product. Bacterial clearance occurred during therapy, and infection did not recur in one case after cessation. Although such therapy was considered, it was not feasible: Pentaglobulin is unavailable in Belgium.

Antibiotic treatment is usually not indicated in *Campylobacter* infections, except in septicemia or persistent disease (12). Our laboratory performs antimicrobial susceptibility testing using disk diffusion. Routine testing includes erythromycin and ciprofloxacin, with susceptibility to azithromycin and clarithromycin inferred from erythromycin susceptibility in accordance with EUCAST recommendations. Additional agents (ampicillin, amoxicillin–clavulanic acid, tetracycline, and gentamicin) are assessed when indicated. EUCAST breakpoints were applied for ciprofloxacin, erythromycin, and tetracycline, while Comité de l'Antibiogramme de la Société Française de Microbiologie (CA-SFM) criteria were used for ampicillin, amoxicillin–clavulanic acid, and gentamicin in the absence of EUCAST and CLSI breakpoints.

The resistance pattern in this case mirrors European surveillance data. Fluoroquinolone resistance in human *C. jejuni* isolates is widespread, with ciprofloxacin resistance at 58%, whereas macrolide resistance remains low (<3%) (13). Recent Belgian reference laboratory data confirm this trend: 67% ciprofloxacin, 42% tetracycline, and 1.8% azithromycin resistance (14).

Beyond resistance dynamics, this case involved recovery of *C. jejuni* from multiple sites over time, providing insight into the infection course. In December 2024, two isolates showed discordant macrolide susceptibility: the hip isolate remained susceptible, consistent with earlier blood isolates, whereas the stool isolate had acquired resistance. This suggests hip dissemination during bacteremia with subsequent intestinal evolution under antibiotic pressure. WGS showed that all isolates belonged to CC-443 (ST-6522), with only two allelic differences between the earliest and latest isolates, consistent with microevolution over time. These findings support persistent infection rather than reinfection and underscore the value of WGS for confirming clonal continuity when phenotypic profiles diverge.

The literature does not provide evidence for an association between the identified *C. jejuni* lineage (CC-443, ST-6522) or its virulence gene repertoire and persistent human infection. ST-6522 has recently been reported as an emerging sequence type among *C. jejuni* isolates from laying hens in China (15), suggesting foodborne transmission rather than chronic adaptation. Overall, these findings point to host factors, not lineage, as the driver of persistence.

Beyond lineage-associated considerations, antimicrobial resistance determinants and their phenotypic expression were also assessed. Fluoroquinolone resistance correlated clearly with genotype, whereas macrolide resistance emerged phenotypically only after azithromycin exposure, despite the presence of resistance-associated mutations in earlier isolates. Discordances between genotypic and phenotypic resistance patterns were noted for β-lactams and tetracyclines. Although the *blaOXA-576* gene was detected, this variant has not been associated with promoter mutations resulting in increased β-lactamase expression, unlike the 67 G→T promoter transversion described for *blaOXA-61* (16). Accordingly, the presence of *blaOXA-576* alone does not confer β-lactam resistance, consistent with phenotypic susceptibility to amoxicillin. Interpretation of these findings is further complicated by the absence of EUCAST and CLSI interpretive breakpoints for β-lactams in *Campylobacter* spp., despite the availability of alternative CA-SFM criteria for selected agents, highlighting the need for cautious interpretation and clinical correlation of phenotypic susceptibility, especially in severe or invasive disease. Similarly, despite the presence of *tet(O*) in all isolates, which usually correlates strongly with tetracycline resistance, the strains remained susceptible. No frameshift mutations were identified that could explain this lack of expression (12).

After 8 weeks of oral gentamicin, stool culture became negative, consistent with gentamicin susceptibility testing and WGS findings. However, persistent PCR positivity—possibly due to residual bacterial DNA or low-level persistence below the culture detection limit—complicated interpretation of ongoing diarrhea. Clinical resolution was ultimately achieved with intravenous meropenem plus azithromycin. Although meropenem susceptibility was not tested phenotypically, WGS showed no carbapenem resistance mechanisms, supporting presumed susceptibility. Given the susceptibility-guided stepwise approach and the initial attribution of persistence to host factors and source control issues, escalation to carbapenem therapy was reserved for a later stage in the clinical course to maintain a narrow-spectrum, targeted treatment strategy and minimize further disruption of the intestinal microbiome.

In conclusion, patients with hypogammaglobulinemia following CD19-directed CAR T-cell therapy face an increased risk of persistent *C. jejuni* infection. In this case, WGS demonstrated that the infection was persistent, excluding reinfection, and underscoring the limitations of relying solely on phenotypic susceptibility for assessing relatedness. Microbiological response was evidenced by culture negativity after an 8-week course of oral gentamicin, despite ongoing PCR positivity, and full clinical resolution was achieved following an additional 2-week course of intravenous meropenem combined with oral azithromycin, without the need for IgA or IgM replacement.

## Data Availability

WGS data can be accessed through study accession no. PRJEB100966.
